# Outcomes of simultaneous left atrial appendage closure in atrial fibrillation patients undergoing transcatheter aortic valve replacement

**DOI:** 10.1097/MS9.0000000000002245

**Published:** 2024-06-17

**Authors:** Hasaan Ahmed, Mahmoud Ismayl, Anirudh Palicherla, Anthony Kashou, Jalal Dufani, Amjad Kabach, Andrew Goldsweig, Nandan Anavekar, Ahmed Aboeata

**Affiliations:** a Department of Medicine, Division of Internal Medicine; b Department of Medicine, Division of Cardiovascular Disease, Creighton University School of Medicine, Omaha, NE; c Department of Cardiovascular Medicine, Mayo Clinic, Rochester, MN; d Department of Cardiovascular Medicine, Baystate Medical Center, Springfield, MA, USA

Atrial fibrillation remains the most prevalent cardiac arrhythmia, with thromboembolism being an often feared complication among those affected. While anticoagulation has been the gold standard of stroke prevention in atrial fibrillation patients, left atrial appendage closure (LAAC) has emerged, serving as a viable alternative in those with non-valvular atrial fibrillation who are unable to tolerate anticoagulation. Despite prior studies noting similar efficacy compared to pharmacotherapy, outcomes of LAAC continue to be a topic of interest with trials ongoing.

Compared to surgical methods, transcatheter aortic valve replacement (TAVR) has become increasingly performed, serving as a practical and less invasive intervention for patients with aortic valve abnormalities. While prior studies have noted promising results in patients with non-valvular atrial fibrillation undergoing TAVR, clinical outcomes in patients with TAVR undergoing concomitant LAAC remain uncertain. Therefore, we performed a meta-analysis to evaluate clinical outcomes in atrial fibrillation patients undergoing simultaneous LAAC and TAVR compared to those undergoing isolated TAVR.

A comprehensive literature search was performed for studies evaluating outcomes of concomitant LAAC and TAVR in atrial fibrillation patients compared to those undergoing isolated TAVR. Outcomes of interest were all-cause mortality, major bleeding, vascular complications, and ischemic stroke. Two evaluators used a two-step screening process for title, abstract, and full-text screening. Any disagreements that arose among reviewers were addressed through extensive discussions. A common-effect model was used to calculate risk ratios (RR) with 95% CIs in R studio for each outcome. Heterogeneity was assessed using the I^2^ test.

A total of three studies were identified consisting of one prospective observational study, one retrospective observational study, and one randomized controlled trial^1–3^. The total study population encompassed 482 patients with atrial fibrillation, of which 239 underwent TAVR with LAAC while 243 underwent isolated TAVR. Simultaneous TAVR with LAAC was associated with a significantly increased risk of major vascular complications (RR 5.44; 95% CI 1.77–16.71 (Fig. 1A)) compared to isolated TAVR. All-cause mortality (RR 0.94; 95% CI 0.64–1.38 (Fig. 1B)), major bleeding (RR 1.18; 95% CI 0.77–1.81 (Fig. 1C)), and ischemic stroke (RR 0.83; 95% CI 0.38–1.80 (Fig. 1D)) were similar between both groups.

**Figure 1:**
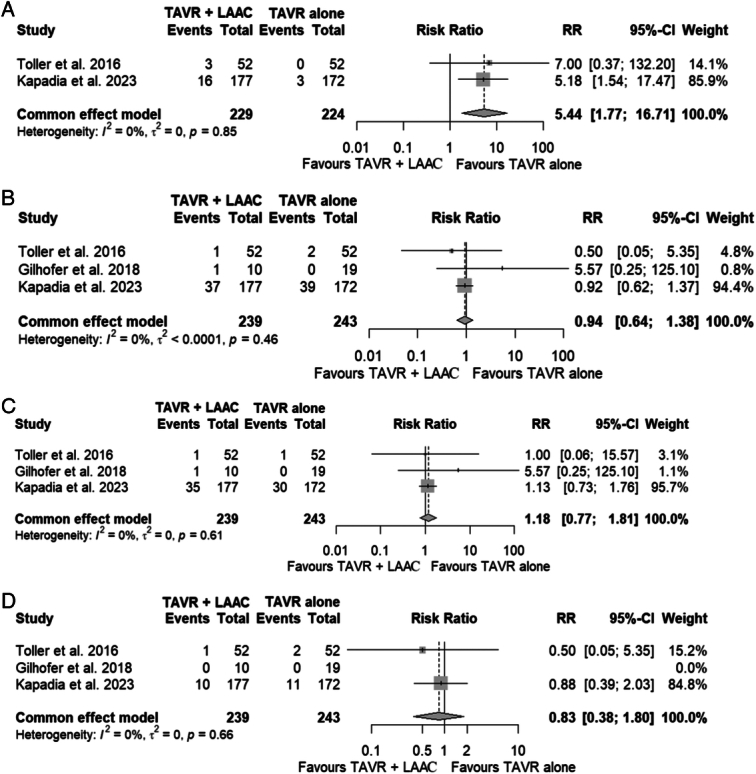
Outcomes of simultaneous left atrial appendage closure in atrial fibrillation patients undergoing transcatheter aortic valve replacement. Funnel plots of outcomes assessed (A) vascular complications; (B) all-cause mortality; (C) major bleeding; (D) ischemic stroke.

Increased vascular complications noted with concomitant TAVR and LAAC are similar to prior studies evaluating clinical outcomes of concomitant LAAC procedures. A retrospective study by Ismayl *et al.* ^4^ found concomitant LAAC and TAVR had higher adjusted odds of stroke (*P*=0.02) and vascular injury (*P*<0.01) compared to isolated LAAC, attributed to multiple invasive interventions which likely propagated the risk of vascular complications occurring, especially since vascular complications are already prominent with TAVR. Increased vascular complications in concomitant TAVR and LAAC are likely multifactorial. Elderly age may have contributed to increased vascular complications as those undergoing LAAC are typically frail and possess contorted vascular anatomy. The use of large-sized delivery sheaths during LAAC may have also amplified the risk of vascular complications occurring. Additionally, variations in operator experience and procedural volume of LAAC likely also contributed to increased vascular complications noted. Further studies are warranted to assess outcomes of concomitant TAVR and LAAC among patients stratified by hospital procedural volume and operator experience.

Similar risks of adverse outcomes encompassing all-cause mortality, major bleeding, and ischemic stroke reflect increased operator knowledge and enhancement of both procedural methods and closure devices of simultaneous TAVR with LAAC. Prior studies have also noted enhanced safety and feasibility of concomitant LAAC with other cardiovascular procedures, as seen by Ismayl *et al.* who noted those undergoing LAAC with a concomitant procedure had similar odds of in-hospital mortality [adjusted odds ratio (aOR) 1.39; 95% CI 0.55–3.54], stroke (aOR 2.09; 95% CI 0.84–3.84), and major bleeding (aOR 1.17; 95% CI 0.72–1.91) compared to those who underwent isolated LAAC^4^. These findings reflect technological advancements across minimally invasive cardiovascular interventions as a whole, in combination with a lower-risk patient population undergoing procedural intervention^4^.

In conclusion, this meta-analysis found simultaneous TAVR and LAAC among atrial fibrillation patients to be associated with an increased risk of vascular complications, with similar outcomes of all-cause mortality, major bleeding, and ischemic stroke noted, compared to those who underwent isolated TAVR. This study may serve as a guide for interventional procedural considerations in patients with atrial fibrillation and aortic valve abnormalities. Further studies are warranted to confirm our findings.

## Ethical approval

Ethical approval was not required for this editorial.

## Consent

Informed consent was not required for this editorial.

## Source of funding

No funding was sought or utilized for this manuscript. This research did not receive any specific grant from funding agencies in the public, commercial, or not-for-profit sectors.

## Author contribution

Conceptualization: H.A., M.I., A.P., A.K., J.D., A.K., A.G., N.A., A.A.; writing—original draft: H.A., M.I., A.P., A.G.; writing—review and editing: H.A., M.I., A.P., A.K., J.D., A.K., A.G., N.A., A.A.; investigation: H.A., M.I., A.P.; supervision: A.G., N.A., A.A.

## Conflicts of interest disclosure

A.G. reports speaking fees from Edwards Lifesciences and Philips and consulting fees from Philips and Inari Medical.

## Research registration unique identifying number (UIN)

Not applicable.

## Guarantor

Hasaan Ahmed.

## Provenance and peer review

Not commissioned, externally peer-reviewed.

## Data availability statement

The data that support the findings of this manuscript are openly available upon reasonable request from the corresponding author.
